# Explosive eye injuries: characteristics, traumatic mechanisms, and prognostic factors for poor visual outcomes

**DOI:** 10.1186/s40779-022-00438-4

**Published:** 2023-01-12

**Authors:** Ying Zhang, Xin Kang, Qiong Wu, Zhong Zheng, Jun Ying, Mao-Nian Zhang

**Affiliations:** 1 Senior Department of Ophthalmology, the Third Medical Center of Chinese PLA General Hospital, Beijing, 100039 China; 2 Department of Ophthalmology, the First Medical Center of Chinese PLA General Hospital, Beijing, 100853 China; 3 Medical Security Center, Chinese PLA General Hospital, Beijing, 100853 China; 4 Information Center, Logistics Support Department, Central Military Commission, Beijing, 100842 China; 5 Information Management Department, Chinese PLA General Hospital, Beijing, 100853 China

**Keywords:** Explosion-related eye injury, Mechanical ocular trauma, Visual outcome, Risk factor

## Abstract

**Background:**

Explosions can produce blast waves, high-speed medium, thermal radiation, and chemical spatter, leading to complex and compound eye injuries. However, few studies have comprehensively investigated the clinical features of different eye injury types or possible risk factors for poor prognosis.

**Methods:**

We retrospectively reviewed all consecutive records of explosive eye injuries (1449 eyes in 1115 inpatients) in 14 tertiary referral hospitals in China over 12 years (between January 2008 and December 2019). Data on demographics, eye injury types, ocular findings, treatments, and factors affecting visual prognosis were extracted from a standardized database of eye injuries and statistically analyzed.

**Results:**

Mechanical ocular trauma accounted for 94.00% of explosion-related eye injuries, among which intraocular foreign bodies (IOFBs) resulted in 55.17% of open globe injuries (OGIs) and contusion caused 60.22% of close globe injuries (CGIs). Proliferative vitreous retinopathy (PVR) was more common in perforating (47.06%) and IOFB (26.84%) than in penetrating (8.79%) injuries, and more common with laceration (24.25%) than rupture (9.22%, *P* < 0.01). However, no difference was observed between rupture and contusion. Ultimately, 9.59% of eyes were removed and the final vision was ≤ 4/200 in 45.82% of patients. Poor presenting vision [odds ratio (*OR*) = 5.789], full-thickness laceration of the eyeball ≥ 5 mm (*OR* = 3.665), vitreous hemorrhage (*OR* = 3.474), IOFB (*OR* = 3.510), non-mechanical eye injury (NMEI, *OR* = 2.622, *P* < 0.001), rupture (*OR* = 2.362), traumatic optic neuropathy (*OR* = 2.102), retinal detachment (RD, *OR* = 2.033), endophthalmitis (*OR* = 3.281, *P* < 0.01), contusion (*OR* = 1.679), ciliary body detachment (*OR* = 6.592), zone III OGI (*OR* = 1.940), and PVR (*OR* = 1.615, *P* < 0.05) were significant negative predictors for poor visual outcomes.

**Conclusions:**

Explosion ocular trauma has complex mechanisms, with multiple eyes involved and poor prognosis. In lethal level I explosion injuries, eyeball rupture is a serious condition, whereas contusion is more likely to improve. In level II injuries, IOFBs are more harmful than penetrating injuries, and level IV represents burn-related eye injuries. PVR is more associated with penetrating mechanisms than with OGI. Identifying the risk predictors for visual prognosis can guide clinicians in the evaluation and treatment of ocular blast injuries.

**Supplementary Information:**

The online version contains supplementary material available at 10.1186/s40779-022-00438-4.

## Background

An explosive blast has sudden and devastating effects and usually causes great casualties [1]. Explosion-related eye injuries are a common cause of morbidities for survivors, whether the injury occurred during wartime, a disaster, or an explosion-related accident during peacetime [2–6]. Explosions rapidly generate impact waves with dramatic power, producing instantaneous peeling, implosion, and hemodynamic effects, and pressure differences (called primary or level I explosion injuries); fragments with high kinetic energy (leading to secondary or level II injuries); huge airflow capable of overturning the human body (leading to level III injuries); and hyperthermic or high-pressure chemical reaction (leading to level IV injuries) [4, 5, 7]. The eyeball is extremely vulnerable to explosive injuries since it is an exposed and incompressible spherical organ full of liquid and rich in vascular networks, with fragile tissues and fine structures. Explosion-related eye injury can cause impaired visual acuity (VA) and even blindness [8]. Owing to the diversity and complexity of explosive injury mechanisms, the difficulty, and uncertainty of diagnosis and treatment are greatly increased in patients with ocular explosive injuries. Thus, identifying the mechanisms and classification of ocular blast injuries in detail is particularly important for timely and accurate treatment to minimize the visual disability rate. The present study was based on a multi-center review of inpatients who experienced explosive injuries. We thoroughly analyzed the mechanisms and classifications of explosion-related ocular injuries, compared the differences among various trauma categories, and assessed meaningful risk factors that affect the visual function outcomes.

## Methods

### Population

This retrospective study was conducted according to the tenets of the Declaration of Helsinki and approved by the ethics committee of the Chinese PLA General Hospital (S2002-074-01). Consecutive medical records of all patients with explosive eye injuries who were admitted to 14 tertiary referral hospitals in China between January 1, 2008 and December 31, 2019 were extracted and retrospectively reviewed. Each case was recorded using a standardized pre-formulated data sheet; the records were maintained in an eye injury database. Ultimately, 1449 eyes from 1115 patients with eye injuries caused by explosions were eligible for analysis.

### Procedures

Detailed information regarding each eye that was injured from an explosion was collected using a standardized data sheet that included patient information (including age, gender, and occupation), a thorough history (including the cause of explosion and timing and nature of the injury), clinical presentation (including initial assessment of visual function following the explosion, if known), and treatments and outcomes (including the final assessment of visual function at discharge or at the end of the follow-up period). In addition, the mechanical globe injury zone and the wound length in the globe wall were recorded. Surgical findings and complete ophthalmological examination results were also retrieved for analysis. The VA data was either best-corrected or pinhole VA. The prognostic factors related to poor prognosis (final VA level) were analyzed. The population was further divided into six groups according to age: 1–6 years (pre-schoolers), 7–12 years (juveniles), 13–18 years (teenagers), 19–39 years (youth adults), 40–59 years (middle-aged adults) and ≥ 60 years (old adults). Individuals ≤ 18 years of age were categorized as adolescents and individuals > 18 years old as adults.

### Definitions

Classification and definition of mechanical eye injury (MEI) were based on the Birmingham Eye Trauma Terminology (BETT) [9]. Open globe injury (OGI) was defined as a full-thickness wound of the eyeball including rupture (blunt force caused damage to the eyeball from the inside out), penetration (entrance wound only in the globe wall), intraocular foreign body (IOFB, retained foreign bodies in the eye) and perforation (simultaneous entrance and exit wounds in the globe wall). The latter three are collectively referred to as laceration (a sharp instrument causes damage to the eyeball from the outside in). Open globe mixture refers to the combined injury of rupture and IOFB or perforation. Close globe injury (CGI) includes those involving contusion (blunt force, although without global rupture), lamellar laceration, and superficial foreign body. Close globe mixture injuries refer to those involving combined contusion and lamellar laceration or superficial foreign body.

The globe injury zones were identified according to the Ocular Trauma Classification System (OTCS) [10]. The OGI zones (location of the full-thickness wound in the globe wall) were defined as within the cornea and the limbus, in the scleral area within 5 mm posterior to the corneoscleral limbus, and extending 5 mm beyond the limbus (zones I, II, and III, respectively). The CGI zones (involved eye tissues) were defined as the ocular surface (limited to bulbar conjunctiva, sclera, and cornea); anterior segment structures and pars plana ciliary; and intraocular structures behind the posterior capsule-lens interface (zones I, II, and III, respectively).

Non-mechanical eye injury (NMEI) included thermal, alkali, and acid burns of the eye. Chronic hypotony was defined as intraocular pressure (IOP) < 8 mmHg after at least 6 months of follow-up. Snellen VA was converted to fractional VA and grouped according to the Ocular Trauma Score (OTS) study group (1 through 5) [11]: no light perception (NLP), light perception (LP)—4/200 (LP—0.02), 5/200–19/100 (0.025—0.19), 20/100–20/50 (0.2—0.4), and ≥ 20/40 (≥ 0.5). Poor VA referred to VA ≤ 4/200 (including the removed eyes). In this study, an increased final VA level (compared with the initial VA level) was defined as final VA improvement and conversely as final VA reduction.

### Statistical analysis

All data were collected from an electronic database and crosschecked for errors. Statistical analyses were performed using SPSS software version 26.0 (IBM, Corp., Armonk, NY, USA). Categorical variables were analyzed using the Chi-square test. Continuous variables were evaluated for normality, and means were compared using a two-tailed *t*-test. Further multiple logistic regression analysis was conducted to predict the independent factors affecting poor vision at the final assessment. *P-*values < 0.05 were considered statistically significant.

## Results

### Demographics characteristics

Approximately 29.96% of the 1115 patients had bilateral ocular trauma (1449 explosion-injured eyes). The mean age of all patients was (28.84 ± 14.52) years (range: 1–76 years; median: 30 years) and the ratio of men to women was 7.64:1. No significant age difference was observed between male and female patients [(28.79 ± 14.11) years vs. (29.23 ± 17.41) years, *F* = 0.107, *P* = 0.744]. Among all eye injuries, 72.91% occurred in adults, and the largest proportion was sustained by the 19–39-year-old group (567 cases, 50.85%) (Additional file 1: Table S1). The proportion of male patients and bilateral eye injuries differed by age group (*χ*^2^ = 17.69, *P* < 0.01; *χ*^2^ = 48.05, *P* < 0.01). The proportion of men was highest in the 13–18-year-old group (92.31%) and the proportion of bilateral eye injuries was highest in the 40–59-year-old group (37.44%) (Additional file 1: Fig S1). The top three occupations reported by patients were workers (34.62%), students (22.06%), and farmers (13.72%). Approximately 46.37% of workers (179 patients) were miners (Additional file 1: Table S1).

### General conditions of the explosion

The top four categories of explosives causing eye injuries were fireworks or firecrackers (35.16%), mine gases (17.13%), detonators (11.93%), and containers (9.87%). The most common complication of systemic explosion damage was limb (13.90%) followed by craniocerebral injury (7.44%). Approximately 1.97% of inpatients were in a coma following the explosion (Additional file 1: Table S1).

### Clinical features of injured eyes

#### Eye injury types

As shown in Table 1, MEI accounted for the vast majority of injuries (94.00%), with simple NMEI accounting for 5.11% of all injuries and both types of mixed injuries present in 70 eyes (4.83%). The proportion of rupture among OGI (28.77%) was lower than that of contusion among CGI (60.22%), although both were generally caused by blunt force (*χ*^2^ = 136.50, *P* < 0.01). The proportion of mixed injuries among OGI (0.98%) was also lower than that in CGI (25.39%; *χ*^2^ = 184.30, *P* < 0.01). IOFB had the highest proportion among OGI (55.17%), while contusions were highest among CGI (60.22%).

**Table 1:** Eye injury types and zones caused by explosion (total of 1449 eyes)^*^ [*n*(%)]

Injury types	No. of eyes	Zone		
		I	II	III
MEI	1362 (94.00)	475 (34.88)	313 (22.98)	574 (42.14)
OGI	716 (52.57)	363 (50.70)	196 (27.37)	157 (21.93)
Rupture	206 (28.77)	64 (31.07)	52 (25.24)	90 (43.69)
Penetration	91 (12.71)	63 (69.23)	22 (24.18)	6 (6.59)
IOFB	395 (55.17)	236 (59.75)	120 (30.38)	39 (9.87)
Perforation	17 (2.37)	0	2 (11.76)	15 (88.24)
Open globe mixture	7 (0.98)	0	0	7 (100.00)
CGI	646 (47.43)	112 (17.34)	117 (18.11)	417 (64.55)
Contusion	389 (60.22)	6 (1.54)	90 (23.14)	293 (75.32)
Lamellar laceration	3 (0.46)	3 (100.00)	0	0
Superficial foreign body	90 (13.93)	90 (100.00)	0	0
Close globe mixture	164 (25.39)	13 (7.93)	27 (16.46)	124 (75.61)
NMEI	144 (9.94)^a^	-	-	-
Thermal burn	95 (65.97)^b^	-	-	-
Alkali burn	23 (15.97)^c^	-	-	-
Acid burn	26 (18.06)^d^	-	-	-

*All percentages in the table were calculated with the number of injured eyes of the higher category to which they belong as the denominator. ^a^74 eyes (5.11%) of simple non-mechanical eye injury, 70 eyes (4.83%) complicated with MEI. ^b^38 eyes (2.62%) of simple thermal burn, 57 eyes (3.94%) complicated with MEI. ^c^19 eyes (1.31%) of simple alkali burn, 4 eyes (0.28%) complicated with (not included in) MEI (alkali burn more severe). ^d^17 eyes (1.17%) of simple acid burn, 9 eyes (0.62%) complicated with (not included in) MEI (acid burn more severe). *MEI* mechanical eye injury, *OGI* open globe injury, *IOFB* intraocular foreign body, *CGI* close globe injury, *NMEI* non-mechanical eye injury

NMEI types differed among mixed-injury cases complicated by MEI (*χ*^2^ = 17.21, *P* < 0.01). In these cases, thermal burn combined with MEI occurred more commonly than alkali or acid burns.

**Fig. 1:**
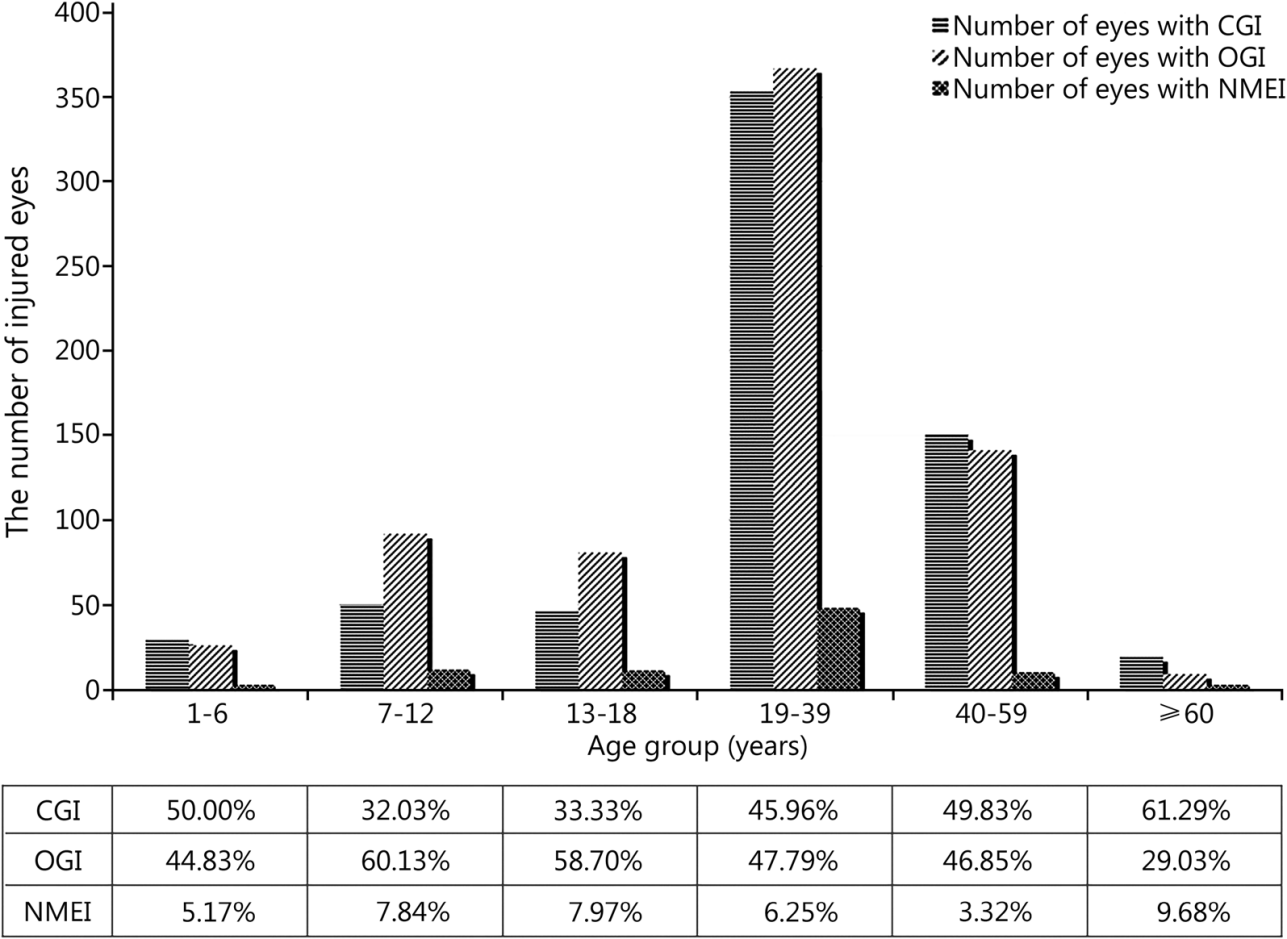
Distribution of the number of injured eyes in each age group and comparison of constituent ratios of different types of ocular trauma among the age groups: the youth adults (19–39 years) having the highest number of traumatic eyes and OGI accounting for the highest proportion in the juveniles (7–12 years). CGI, OGI and NMEI within each age group. OGI open globe injury, CGI close globe injury, NMEI non-mechanical eye injury

Figure 1 shows the distribution of the proportions of eye injury types according to age group (*χ*^2^ = 29.67, *P* < 0.001). The absolute number of eyes among all three types of ocular trauma was the largest in the 19–39-year-old group. The proportions of CGI (61.29%) and NMEI (9.68%) were highest in the ≥ 60-year-old group; while that for OGI was highest in the 7–12-year-old group (60.13%). In addition, the OGI composition ratio was higher in patients aged 7–39 years (540/816, 66.18%) compared with that in patients aged < 7 years and ≥ 40 years (176/299, 58.86%; *χ*^*2*^ = 5.09, *P* < 0.05).

#### Zone of mechanical ocular trauma

In all eyes with MEI, the most affected zone was zone III (42.14%, 574/1362), followed by zones I (34.88%, 475/1362) and II (22.98%, 313/1362). The zone distribution differed between OGI and CGI (*χ*^2^ = 267.45, *P* < 0.01), in all types of OGI (*χ*^2^ = 274.775, *P* < 0.001) and in CGI (*χ*^2^ = 579.66, *P* < 0.001). OGI typically involved zone I while CGI involved zone III. Among OGI, no statistical difference in zoning was observed between penetration and IOFB (*χ*^2^ = 2.91, *P* > 0.05) injuries. The rupture was most common in zone III while laceration preferentially involved zone I (*χ*^2^ = 93.27, *P* < 0.01). Similar to rupture, contusion generally involved zone III (Table 1).

### Ocular findings

The ocular findings are shown in Table 2. Among ocular blast injuries, 20.57% were associated with eyelid damage, and the incidence of superficial foreign body injuries and IOFB injuries was as high as 31.61% and 27.54%, respectively. Explosion-related eye injuries resulted in high proportions of the anterior chamber (hyphema, 27.88%) and vitreous hemorrhage (VH, 40.10%); the most common tissue injury was traumatic cataract (47.48%). The proportions of other eye tissue injuries exceeding 10% included optic axis corneal opacity, iridodialysis, lens capsule breach, lens dislocation, retinal breaks, retinal detachment (RD), and proliferative vitreous retinopathy (PVR).

**Table 2:** Ocular findings in 1449 injured eyes following explosion [*n*(%)]

Findings	No. of eyes
Eyelid injuries	298 (20.57)
Lacrimal canaliculus rupture	25 (1.73)
Orbital fracture	30 (2.07)
Obital hypertension	5 (0.35)
Location of foreign bodies	
Intraorbital	92 (6.35)
Superficial	458 (31.61)
Intraocular	399 (27.54)
Corneal damage	
Whole corneal opacity	71 (4.90)
Optic axis corneal opacity	148 (10.21)
Corneal ulcer	19 (1.31)
Cornea blood staining	16 (1.10)
Anterior chamber	
Abnormal depth	37 (2.55)
Hyphema	404 (27.88)
Hypopyon	15 (1.04)
Vitreal hernia	24 (1.66)
Iris	
Iridodialysis	166 (11.46)
Iris defect	69 (4.76)
Iris synechia	117 (8.07)
CBD	24 (1.66)
IOP	
Traumatic glaucoma	114 (7.87)
Chronic hypotony	58 (4.00)
Lens	
Lens capsule breach	173 (11.94)
Lens dislocation	159 (10.97)
Traumatic cataract	688 (47.48)
VH	581 (40.10)
Retina	
Retinal breaks	200 (13.8)
RD	194 (13.39)
Retinal defect	17 (1.17)
Retinomalacia	12 (0.83)
Subretina hemorrhage	78 (5.38)
Choroid	
Choroid detachment	52 (3.59)
Suprachoroidal hemorrhage	31 (2.14)
Choroidal laceration	35 (2.42)
Choroidal defect	8 (0.55)
TON	124 (8.56)
Endophthalmitis	50 (3.45)
Sympathetic ophthalmia	1 (0.07)
PVR	178 (12.28)

*CBD* ciliary body detachment, *IOP* intraocular pressure, *VH* vitreous hemorrhage, *RD* retinal detachment, *TON* traumatic optic neuropathy, *PVR* proliferative vitreous retinopathy

Table 3 shows the differences in the proportions of traumatic cataracts, VH, RD, and PVR among different types of explosion-related ocular trauma. Traumatic cataracts, VH, RD, and PVR all occurred in significantly higher proportions in OGI compared with CGI (*P* < 0.01). Contusion had a much lower incidence of VH and RD compared with those of rupture (*P* < 0.01); no significant difference was observed in the incidence of traumatic cataracts or PVR (*P* > 0.05).

**Table 3:** Percentage comparisons of main ocular findings in different eye types [*n*(%)]

Types of eye injury	Traumatic cataract		VH		RD		PVR	
General classification								
OGI	460 (64.25)	*χ*^2^ = 117.55, *P* < 0.01	391 (54.61)	*χ*^2^ = 88.15, *P* < 0.01	156 (21.79)	*χ*^2^ = 70.33, *P* < 0.01	141 (19.69)	*χ*^2^ = 58.30, *P* < 0.01
CGI	225 (34.83)		190 (29.41)		38 (5.88)		37 (5.73)	
Blunt force injuries in OGI and CGI								
Rupture	86 (41.75)	*χ*^2^ = 0.05, *P* = 0.82	94 (45.63)	*χ*^2^ = 7.95, *P* < 0.01	49 (23.79)	*χ*^2^ = 42.05, *P* < 0.01	19 (9.22)	*χ*^2^ = 1.38, *P* = 0.24
Contusion^a^	225 (40.83)		190 (34.48)		38 (6.90)		37 (6.72)	

^a^551 eyes including 389 eyes of simple contusion and 162 eyes of close globe mixture complicated with contusion. *OGI* open globe injury, *CGI* close globe injury, *VH* vitreous hemorrhage, *RD* retinal detachment, *PVR* proliferative vitreous retinopathy

Furthermore, the occurrence of PVR was particularly noticeable in perforating (47.06%, 8/17) and IOFB (26.84%, 106/395) injuries compared with that of penetrating injuries (8.79%, 8/91; *χ*^2^ = 18.09, *P* < 0.01), and was more commonly observed in laceration (24.25%, 122/503) than in ruptures (9.22%, 19/206; *χ*^2^ = 20.73, *P* < 0.01). Among all types of OGI or laceration, the PVR incidence differed significantly (*χ*^2^ = 43.24, *P* < 0.01; *χ*^2^ = 18.09, *P* < 0.01).

### Clinical treatments

Surgery was performed in 1055 eyes (72.81%, 1055/1449), of which 451 eyes (42.75%, 451/1055) were operated on within 24 h of the explosion. The average number of operations was (1.58 ± 0.92), and 435 eyes (41.23%, 435/1055) underwent ocular surgeries more than twice (up to 11 times). Intraocular operations were performed in 816 eyes (56.31%, 816/1449), with an average of (1.54 ± 0.82) times, and two or more times (up to 9) in 324 eyes (39.71%, 324/816). Vitrectomy was performed in 363 eyes (25.05%, 363/1449), and at least twice in 62 eyes (17.08%, 62/363). Significant statistical differences were observed in the intraocular surgery rates among the different age groups (*χ*^2^ = 17.32, *P* < 0.01). The top three age groups that underwent intraocular surgery were 1–6-year- (69.81%, 37/53), 7–12-year- (66.67%, 88/132), and 13–18-year-old (63.24%, 74/117) groups.

### Outcomes

According to the outcome at discharge or follow-up, 73 eyes (5.04%, 73/1449) remained filled with silicone oil, 179 eyes (12.35%, 179/1449) had an intraocular lens, 351 eyes (24.22%, 351/1449) had no lens, 41 eyes (2.83%, 41/1449) had permanent macular lesions, 96 eyes (6.63%, 96/1449) had optic nerve atrophy, and 34 eyes (2.35%, 34/1449) had phthisis bulbus. A total of 139 eyes (9.59%, 139/1449) were removed, among which 101 eyes were enucleated and 38 were eviscerated; 60 eyes (43.17%, 60/139) were removed within 48 h after the explosive injury.

**Fig. 2:**
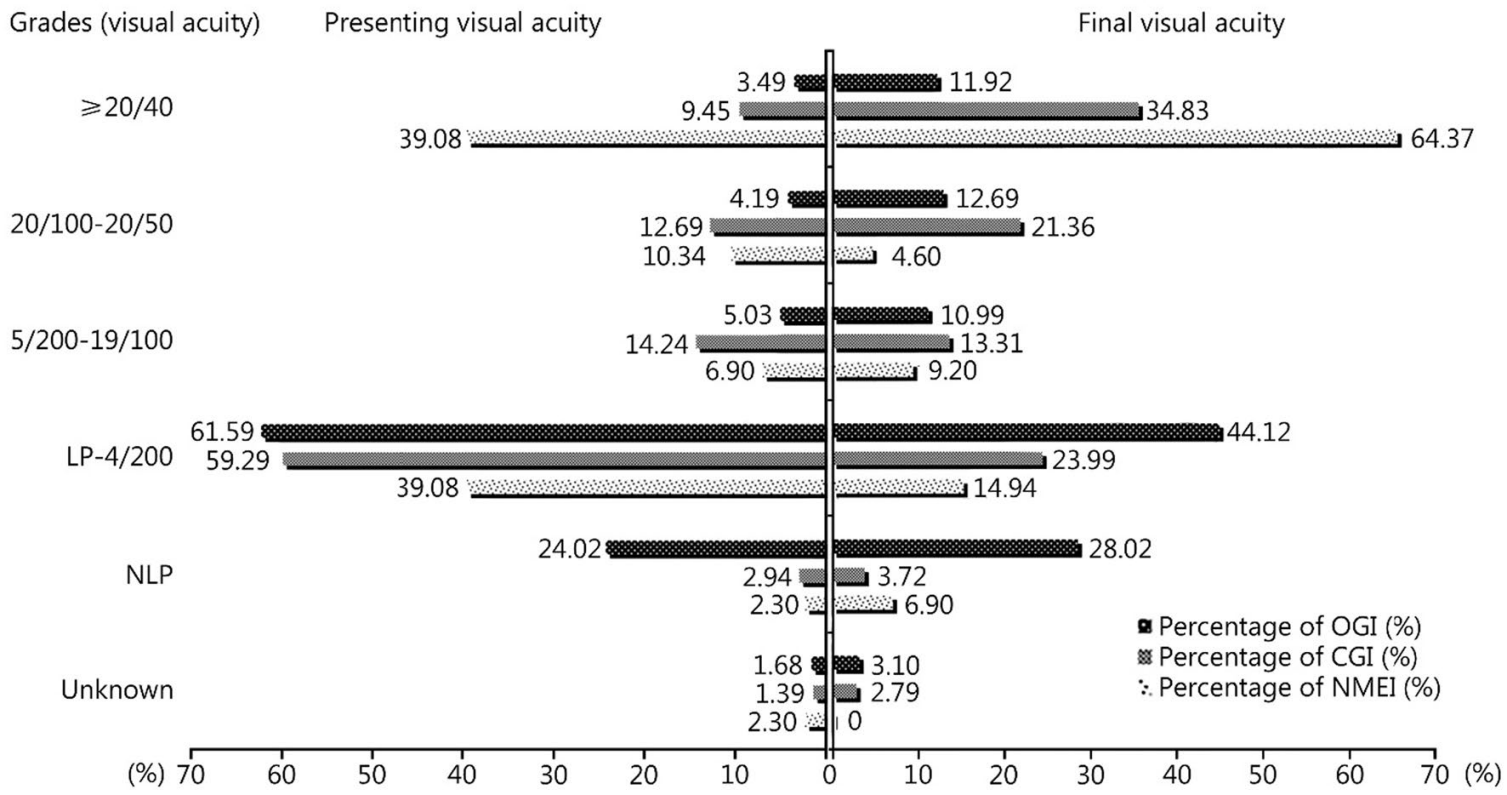
Comparison between the presenting and final visual acuity in proportion (%) of different eye injury types (OGI, CGI, and NMEI). Grade of VA was grouped according to the Ocular Trauma Score. OGI open globe injury, CGI close globe injury, NMEI non-mechanical eye injury, VA visual acuity, LP light perception, NLP no light perception

The presenting and final VA levels are summarized in Table 4. The final vision was ≤ 4/200 in 45.82% of patients (including NLP in 14.56%). The distribution of VA grading changed significantly (*χ*^2^ = 278.31, *P* < 0.01). This comparison differed by injury type (Fig. 2). OGIs had the highest proportions of patients with poor VA (initial or final) (*χ*^2^ = 138.31; *χ*^2^ = 231.25, *P* < 0.001). The presenting and final VA levels in patients with NMEI were generally better than that in patients with OGI and CGI.

**Table 4:** Presenting and final visual acuity of eyes following explosion^*^ [*n*(%)]

VA	Presenting	Final	*χ*^2^	*P*
20/40 or better	120 (8.28)	358 (24.71)	278.31	< 0.01
20/100–20/50	121 (8.35)	224 (15.46)		
5/200–19/100	134 (9.25)	165 (11.39)		
LP–4/200	858 (59.22)	453 (31.26)		
NLP	193 (13.32)	211 (14.56)		

*The presenting VA of 23 eyes (1.59%) and the final VA of 38 eyes (2.62%) was unknown. *VA* visual acuity, *LP* light perception, *NLP* no light perception (including the removed eyes)

Overall, compared with the initial VA level, the final VA improved in 60.39% of eyes, remained unchanged in 34.24%, and deteriorated in 5.73%. Table 5 shows how different eye injury types compared in terms of poor VA, changes in VA, and enucleation rate. The proportions of patients with presenting and final VA levels ≤ 4/200 and enucleation rates were significantly higher in OGI than that in CGI and NMEI (*χ*^2^ = 138.31, *χ*^2^ = 231.25, and *χ*^2^ = 108.29 respectively, *P* < 0.001). And the VA improvement rate was significantly lower in OGI than that in CGI and NMEI (*χ*^2^ = 91.10, *P* < 0.001). The rupture was associated with lower VA improvement and reduction rates compared with laceration, although higher proportions of poor VA (presenting and final) and higher enucleation rates (*χ*^2^ = 14.96, *χ*^2^ = 10.38, *χ*^2^ = 5.43, *χ*^2^ = 9.41 and *χ*^2^ = 39.41 respectively, *P* < 0.05). IOFB and perforating injuries were associated with higher proportions of poor VA (presenting and final) than that in penetrating injuries and lower VA improvement rates (*χ*^2^ = 21.36, *χ*^2^ = 34.71 and *χ*^2^ = 10.42 respectively, *P* < 0.01). No significant difference was found in the final VA reduction (*χ*^2^ = 1.94, *P* = 0.340) and enucleation rates (*χ*^2^ = 4.92, *P* = 0.086) among the three types of laceration. Rupture was associated with higher poor presenting and final VA and enucleation rates than that for contusion (*χ*^2^ = 38.13, *χ*^2^ = 112.41 and *χ*^2^ = 148.11 respectively, *P* < 0.001), although significantly lower VA improvement and reduction rates (*χ*^2^ = 58.68, and *χ*^2^ = 18.43 respectively, *P* < 0.001).

**Table 5:** Comparisons of percentage of VA conditions among different eye injury^*^ [*n*(%)]

Types of eye injury	Presenting VA ≤ 4/200		Final VA ≤ 4/200		Enucleation of eyeball		Final VA improvement		Final VA reduction	
General classification										
OGI	613 (85.61)	*χ*^2^ = 138.31, *P* < 0.001	471 (65.78)	*χ*^2^ = 231.25, *P* < 0.001	127 (17.74)	*χ*^2^ = 108.29,* P* < 0.001	345 (48.19)	*χ*^2^ = 91.10, *P* < 0.001	46 (6.42)	*χ*^2^ = 2.53, *P* = 0.269
CGI	402 (62.23)		179 (27.71)		11 (1.70)		475 (73.53)		36 (5.57)	
NMEI^a^	36 (41.38)		14 (16.09)		1 (1.15)		53 (60.92)		2 (2.30)	
Blunt and sharp force injuries in OGI^b^										
Rupture	186 (90.29)	*χ*^2^ = 5.43, *P* = 0.025	153 (74.27)	*χ*^2^ = 9.41, *P* = 0.002	66 (32.04)	*χ*^2^ = 39.41,* P* < 0.001	76 (36.89)	*χ*^2^ = 14.96, *P* < 0.001	10 (4.86)	*χ*^2^ = 10.38, *P* = 0.001
Laceration	420 (83.50)		313 (62.23)		61 (12.13)		266 (52.88)		36 (7.07)	
Sharp force injuries in OGI										
Penetrating	61 (67.03)	*χ*^2^ = 21.36, *P* < 0.001	32 (35.16)	*χ*^2^ = 34.71, *P* < 0.001	6 (6.59)	*χ*^2^ = 4.92,* P* = 0.086	61 (67.03)	*χ*^2^ = 10.42, *P* = 0.005	4 (4.40)	*χ*^2^ = 1.94, *P* = 0.340
IOFB	342 (86.58)		270 (68.35)		51 (12.91)		199 (50.38)		30 (7.59)	
Perforating	17 (100.00)		11 (64.71)		4 (23.53)		6 (35.29)		2 (11.76)	
Blunt force injuries in OGI and CGI										
Rupture	186 (90.29)	*χ*^2^ = 38.13, *P* < 0.001	153 (74.27)	*χ*^2^ = 112.41, *P* < 0.001	66 (32.04)	*χ*^2^ = 148.11,* P* < 0.001	76 (36.89)	*χ*^2^ = 58.68, *P* < 0.001	10 (4.86)	*χ2* = 18.43, *P* < 0.001
Contusion^c^	376 (68.24)		173 (31.40)		11 (2.00)		407 (73.87)		28 (5.08)	

*Excluding the eyes whose visual acuity was unknown; comparison by Chi-square test. ^a^87 eyes of non-mechanical injury excluding 57 eyes of thermal burn complicated with MEI. ^b^Excluding 7 eyes of open globe mixture. ^c^551 eyes including 389 eyes of simple contusion and 162 eyes of close globe mixture complicated with contusion. *VA* visual acuity, *OGI* open globe injury, *CGI* close globe injury, *NMEI* non-mechanical eye injury, *IOFB* intraocular foreign body

### Prognostic indicators for poor final VA

As shown in Table 6, logistic regression analysis revealed the independent risk predictors of poor final VA (≤ 4/200). The final VA had a significantly higher probability of being worse than 4/200 if the injured eyes were characterized according to an initial presenting VA of less than 4/200 on admission [odds ratio (*OR*) = 5.789, *P* < 0.001], being classified as certain types of eye injury [such as IOFB (*OR* = 3.510, *P* < 0.001), NMEI (*OR* = 2.622, *P* < 0.001), rupture of globe (*OR* = 2.362, *P* = 0.002) and contusion of eyeball (*OR* = 1.679, *P* = 0.017)], some clinical characteristics [such as full thickness laceration of the eyeball was at least 5 mm (*OR* = 3.665, *P* < 0.001), hemorrhage pooled in the vitreous cavity (*OR* = 3.474, *P* < 0.001), the retina was detached (*OR* = 2.033, *P* = 0.005), detachment or dissociation of the ciliary body developed (*OR* = 6.592, *P* = 0.020) and traumatic optic neuropathy (TON) occurred (*OR* = 2.102, *P* = 0.003)], the wound of OGI was in zone III (*OR* = 1.940, *P* = 0.034), complication with endophthalmitis (*OR* = 3.281, *P* = 0.006) and development of secondary PVR (*OR* = 1.615, *P* = 0.049) after explosive eye injuries.

**Table 6:** Multivariate analysis of independent risk factors for final VA^a^ of eyes following explosive injury^*^

Factor	*P*	*OR*	95%CI
Poor presenting VA^a^	0.000	5.789	4.004–8.368
Full thickness laceration of eye ball ≥ 5 mm	0.000	3.665	2.343–5.732
VH	0.000	3.474	2.530–4.769
IOFB	0.000	3.510	2.290–5.379
NMEI	0.000	2.622	1.573–4.368
Rupture	0.002	2.362	1.366–4.0085
TON	0.003	2.102	1.281–3.449
RD	0.005	2.033	1.238–3.337
Endophthalmitis	0.006	3.281	1.399–7.696
Contusion	0.017	1.679	1.096–2.572
CBD	0.020	6.592	1.348–32.242
OGI	0.034	1.940	1.053–3.576
PVR	0.049	1.615	1.001–2.605
CGI	0.592	0.891	0.583–1.360
Craniocerebral injury	0.663	1.107	0.701–1.750
Hyphema	0.757	0.950	0.687–1.314
Perforating	0.764	0.827	0.240–2.855

*Excluding 38 eyes whose final VA was unknown. ^a^Poor VA indicated the VA was ≤ 4/200. *OR* odds ratio, *VA* visual acuity, *IOFB* intraocular foreign body, *NMEI* non-mechanical eye injury, *TON* traumatic optic neuropathy, *RD* retinal detachment, *CBD* ciliary body detachment, *OGI* open globe injury, *PVR* proliferative vitreous retinopathy, *CGI* close globe injury

## Discussion

Explosions can result in various degrees of injuries distributed across a wide range due to different mechanisms initiated by the blast [1, 7]. Generally, injuries caused by explosion involve significantly more binocular trauma (29.96% in the present study; 3.33–72.91% in previous reports [4–8, 12, 13]) compared with those related to ordinary causes (0–2.13%) [14–17]. The number of eyes involved seems to be related to the type and power of the explosives, for example, 3.33–16.32% for firework-related injuries [6, 12] and 72.91% for injuries related to explosive military ammunition [13]. This explosive difference also leads to different types of MEI. In this study, OGI (52.57%, 716/1362) was more common than CGI (47.43%, 646/1362). In previous studies, the OGI ratio (56.60–81.25%) was much higher than the CGI ratio (18.75–43.4%), some of which might be related to the fact that most of the explosives involved were bombs, weapons, and mines [2, 6–8, 12, 13]. Conversely, the proportion of CGI (64.50–67.34%) was higher than that of OGI (26.53–35.5%) in fireworks-related injuries [6, 12]. In cases of ocular trauma derived from common causes, CGI was more frequent than OGI [14, 18, 19] with a few exceptions [20]. The causes and sites of explosion injuries, positional relationship between the injured and the explosives, differences in national or regional working conditions and living standards, and statistical range of eye injury (i.e., simple eye appendage injury or not) are possible reasons for the observed differences. Frimmel et al. [21] reported that chemical (45%) and thermal burns (26%) (level IV injuries) were far more common than MEI in fireworks-related eye injuries, probably because the majority of injuries in this study were eyelid and conjunctiva burns and did not involve the eyeball.

The ocular trauma classification can be analyzed and interpreted based on the mechanism of explosive injury. Primary (level I) explosion injuries develop when the explosive shockwaves produce pressure differences followed by an implosion effect in the eyeball. The maximum IOP during an explosion can reach a peak of 0.29 MPa (2175 mmHg) at 1.63 ms, which is two times larger than the physiological IOP in healthy eyes [22]. Karimi et al. [23] suggested that IOP could reach as high as 15,000–17,000 mmHg if ground blast reinforcement effects were considered in their three-dimensional (3D) finite element model of ocular blast injury. Blunt force crushing the eye can lead to contusion (a type of CGI) followed by rupture (a type of OGI) of the eyeball when its pressure limit is exceeded. In our study, contusion and rupture accounted for 41.06% of all injured eyes and contusion was more common than rupture, similar to some literatures [5, 14]; however, bomb explosions cause more ruptures than contusion injuries [7]. Other factors that influence the blast effect include the peak overpressure achieved, its duration, distance from the explosion, and whether the eyes were pointed towards the explosion or not. Secondary (level II) explosion injuries develop when fragments from the explosives or exogenous fragments pushed by the explosion tear the cornea or sclera (laceration of OGI, including IOFB, penetration and perforation), cut the eyeball surface and wall (lamellar laceration or superficial foreign body, types of CGI) or cause blunt contusion or rupture. Regarding action time, the speed of the primary blast wave was considerably higher and could reach the eyeball sooner than the debris driven by it. If the fragments reached the eyeball, the injury intensity was dramatically increased [24]. Therefore, level II explosion injuries are secondary to level I injuries in mechanism, order, or both. Moreover, level II explosion injuries cannot exist independently in theory and are considered compound injuries of different forms and degrees. In our study, IOFB (27.26%, 395/1449) was the most common type of eye injury, and other types accounted for only a small fraction. When specifically associated with war and weapons use, the proportion of IOFB among previous reports also differed (9.33–54.20%) [2, 7, 13, 20, 25], and its proportion in OGI was as high as 46.32–95.70% [13, 26] (compared with 55.17% in our study). In ocular trauma from common causes, the proportion of IOFB (1.0–21.3%) was lower than that of explosive injuries [14, 16, 27]. In a grade III explosion injury, the human body is overturned by the explosion airflow or is hit by other objects. Our data were consistent with the classification for explosive injuries. Contusion, rupture, and IOFB were the injury types most closely associated with level I and level II explosion injuries.

The ocular injury zone location according to OTCS (in which the eyeball tissues are divided according to repair ability and accessibility) is closely related to the prognosis of visual function. The ocular wall involved in the zone I can be repaired, whether the injury is an OGI or CGI. Because the cornea is a refractive medium, corneal damage affects VA, which can be improved by certain treatments, such as corneal transplantation. Zone II involves the iris, ciliary body tissue, and lens, which affect the pupil and IOP. Intraocular lenses can make up for the abnormal loss of function of the natural lens. The retina is the nerve layer responsible for eye visual perception and transmission, and is the key tissue involved in zone III injuries, which are difficult to repair. Low IOP caused by ciliary body injury and visual function damage caused by retinal injury are the most stubborn and difficult injuries to cure and improve. A poor prognosis is generally associated with zone III, followed by zones II and I [28]. In our study, the ranking of ratios of eyes injured by explosives was zone III > zone I > zone II among all cases of MEI; OGI zone I and CGI zone III were the most affected. Rupture and contusion were among the three most common types, and both generally involved zone III. However, the location of general eye injuries or CGI from general causes has been most often reported to be in zone I [3, 19] or II [27, 29], which is in contrast with our results. This comparison shows to some extent that explosion-related eye injuries predominantly involve the posterior segment (zone III) of the eye and are therefore more serious than general eye injuries from general causes. Meanwhile, it also shows that ruptures and contusions, are mainly caused by level I ocular explosion injury, and are the most important, non-negligible, and destructive types of blast trauma. A 3D fluid-structure interaction model of the human eye simulating the deformation of the eye components owing to primary blast injury showed that the amount of von Mises stresses (ability to resist deformation) on different ocular tissues was relevant to their mechanical and material properties. In terms of stress and strain, the eye components ranked from high to low were the sclera, ciliary body, cornea, lens, iris, retina, muscle, optic nerve, aqueous body, and vitreous body [30]. Although the optic nerve and retina (especially the macula) are located in zone III (the rearward segment of the eyeball), they are most likely disrupted by even a small stress of strain leading to blindness. Conversely, the lowest stresses on the vitreous and aqueous humor, the root of the incompressible sphere, indirectly lead to global wall rupture, and the stress is the highest on the sclera and lowest on the extraocular rectus muscles. Another computational eye-specific model also indicated that the highest stresses and strains were near the rectus muscle insertions into the sclera [31]. This finding is likely the root cause for rectus insertions into the sclera, which is the most vulnerable site of globe rupture.

In our study, traumatic cataracts, VH, RD, and PVR all occurred more in OGI than in CGI, which indicates that, regardless of whether the sharp objects directly penetrate the eyeball or rupture it indirectly, the global structural integrity destructions indicate that the local or overall intraocular tissues undergo more severe, extensive, and diverse types of damage. No statistically significant difference was observed in the proportion of traumatic cataract in contusions and ruptures, which supports that zone II (where the lens is located) is equally involved in both types of ocular trauma by blunt force. Tears of the eyeball wall along with the adjacent uveal vascular networks can inevitably lead to intraocular hemorrhage including VH which, then, was more common in rupture than contusion. Among contusion injuries, RD only developed when the blunt impact from the shockwave produced a strain in the retina high enough to exceed the specified threshold for RD [32]. Obviously, rupture can directly cause retinal/choroidal break, and the retina also can be pulled away by the lost or prolapsed vitreous body or vacuumed out because of the significant loss of intraocular contents and pressure. While no difference was observed in the proportion of PVR between contusions and ruptures, PVR was significantly more common in lacerations (especially perforating and IOFB injuries) than in ruptures. Therefore, we propose a bold hypothesis: the probability of PVR caused by blunt force is not necessarily related to whether the eyeball is broken or not, and the mechanism of the foreign object penetrating the eyeball significantly promotes the PVR occurrence and development.

At the final follow-up, 9.59% of eyes had been removed following explosive injuries in our study. In previous reports, this percentage was only 0.61–10.00% for general or firework-related eye injuries [4–6, 12, 21, 28, 33–36], although it was as high as 11.90–34.46% for injuries caused in war or by bomb or mine blasts [2, 8, 13, 26, 37, 38]. The NLP in our study was 13.32% for the presenting VA and 14.56% for the final VA, which was significantly lower than the values provided in previous reports (initial NLP range 18.7–53.1%) [3, 4, 7, 8, 13, 28, 37] or the final NLP in 14.04–39.00% [3, 4, 8, 12, 28, 36, 37] of injuries related to firecrackers, bomb or mine explosions, war or weapon use, or OGI. This difference is probably related to the fact that our study included data for various causes of explosive injuries and types of eye injuries from mild to heavy, whereas Feng et al. [36] reported exclusively on eyes with severe explosive injuries that required vitrectomy. Additionally, the high proportion of low VA observed in our study [LP - 4/200: 59.22% (initial); 31.26% (final)] as well as in previous reports [LP - hand moving (HM)/ counting fingers (CF): 34.00–56.47% (initial) [3, 4, 8, 28, 37]; 18.0–55.6% (final) [3, 4, 8, 13, 28, 37]] should not be ignored. The challenges of eye trauma treatment require increased clinical focus. In previous studies, after treatment, final VA level ≥ 20/40 was observed in 13.00–42.11% of patients [3, 4, 8, 12] (24.71% in ours); the VA improved in 31.0–67.3% of patients [3, 5, 6, 26, 37] (60.39% in ours). OGIs were often associated with worse visual prognosis in relation to highly lethal explosives, which is consistent with our comparison of presenting and final VA levels among injury types. And when compared with CGI, the proportion of patients with a final VA < 0.1 was higher [3] and the mean VA was lower [13]. We further compared the classification of different ocular trauma mechanisms based on the proportions of patients with VA levels ≤ 4/200, VA reduction and enucleation rates, and inferred the following. (1) In OGI, the percentages of low VA levels and enucleation rates for ruptures caused by blunt force trauma are higher than those for lacerations caused by sharp objects, although the percentage of VA change or improvement after treatment is smaller among ruptures. Briefly, the harm caused by ruptures is greater than that caused by laceration and yet VA differentiation in some laceration cases is poor and not optimistic. (2) Among the three types of laceration by sharp objects, IOFB and perforating injuries can cause in a higher proportion of patients with low vision and a lower proportion of patients with VA improvement compared with penetrating injuries; however, the three types do not differ in final VA reduction and enucleation rates. (3) Rupture as a type of OGI and contusion as a type of CGI are both caused by blunt force. The proportion of patients with low vision and the enucleation rate after a rupture injury is higher, and the final VA changes (whether improvement or reduction) is lower. All these prove once again that a contusion occurs before the eyeball is subjected to instantaneous shock and compression of the blunt force beyond the threshold that the eyeball wall can withstand, and once this threshold is exceeded, a rupture injury occurs. Based on the results of this study, recovery from a high-severity of globe rupture with extensive ocular tissue damage is difficult. Few studies have compared visual function and prognosis of ocular trauma according to the injury mechanism. However, based on our analysis, determining the mechanism of injury and accurately classifying the ocular trauma contributes to the prioritization of ocular trauma treatment, injury evaluation, and outcome prediction.

As far as predictors of poor final VA, initial VA is generally acknowledged as a key factor that affects final vision status [3, 14, 28, 39–42], which is consistent with our results. To some extent, initial VA after ocular trauma reflects the severity of intraocular tissue damage in most eye injuries. In terms of eye injury type, IOFB (mainly associated to level II explosion injury), rupture, contusion (resulting from level I injuries) and NMEI (level IV injuries) were independent risk factors for poor vision. Indeed, these findings make it evident that explosion injuries cause great harm to eyeballs. The rupture severity discussed above is an important risk factor of poor vision, which is supported by the literature [5, 26, 38, 41]. Contusion, although not a scoring factor for low vision in OTS (a simplified predictive tool for ocular trauma) [42], showed significance in this study probably owing to the inclusion of explosion injuries, and zone III (posterior segment) accounting for the vast majority of injuries (75.32%). Indeed, injuries located in zone III were recognized as an independent risk factor for low vision in this study and many others [4, 38, 41]. Consistent with our results, full-thickness laceration of the eyeball ≥ 5 mm (or larger wounds) [26, 40, 42], VH [19, 40, 41], RD [3, 5, 40, 41], TON [5], endophthalmitis [39, 42] and PVR [36] were identified as risks factors for poor VA in some previous analyses. Regarding eye structure and function, damage that involves the posterior segment is not easily repaired. Involvement of the retina or choroid was revealed as predictive factor of poor VA in previous reports [5, 18, 27, 36]. Few studies have been conducted on ciliary body detachment (CBD), as most case analyses do not include this observation. In addition to initial VA, rupture, endophthalmitis and RD, the OTS includes perforation and relative afferent pupillary defect (RAPD) [11]. Because of the small number of perforating cases, few effective statistical analyses can be conducted, which may be the reason it has not been identified as an independent risk factor in many studies, including ours. In certain instances, especially of OGI, RAPD cannot be examined, evaluated and recorded after injury, and therefore was not included in this study. The OTS has general significance as a reference for the evaluation of visual function after ocular trauma, although the particularities of explosive ocular injury should be considered due to its special characteristics compared with conventional eye injuries. The predictive value of these risk factors is useful for patients counseling as well as managing expectations and guiding clinical decisions.

## Conclusions

This multi-center review revealed that explosions cause high rates of bilateral ocular trauma, complex injury conditions and are associated with increased incidence of zone III (posterior segment) involvement and poor prognosis. Eyeball contusions and ruptures associated with primary (level I) explosion injuries, lacerations (especially those caused by IOFB) secondary (level II) to explosion-related injuries, and NMEI related to grade IV explosive injury (burns) were the main mechanisms identified in our study. Rupture injuries tend to predict a worse prognosis and lower probability of recovery. Compared with pure penetrating injuries, the harm caused by IOFB and perforation is more severe. Moreover, we suggest that PVR caused by blunt force is not necessarily related to eyeball rupture, and the mechanism of penetration by a foreign object into the eyeball, especially by IOFBs or direct penetrating of the retinal/choroid, promotes the PVR occurrence and development. Worse presenting VA, larger-sized eyeball wall wound, clinical signs (VH, TON, RD, CBD, endophthalmitis and zone III OGI), injury type (IOFB, rupture and contusion) and PVR development were associated with worse visual outcomes among injured eyes following an explosion. Future studies that include large and homogeneous case and control populations are required to evaluate the prognostic scoring system of eye blast injuries among different types of eye injuries caused by general causes.

## Supplementary Information

**Additional file 1: Table S1.** General features of the explosive eye injuries (total of 1115 patients).** Fig. S1.** Distribution of the number of patients and the proportions of male patients and bilateral eye injuries in different age groups.

## Data Availability

Not applicable.

## Abbreviations

3D: Three-dimensional
BETT: Birmingham eye trauma terminology
CBD: Ciliary body detachment
CF: Counting fingers
CGI: Close globe injury
HM: Hand moving
IOFB: Intraocular foreign body
IOP: Intraocular pressure
LP: Light perception
MEI: Mechanical eye injury
NLP: No light perception
NMEI: Non-mechanical eye injury
OGI: Open globe injuries
OR: Odds ratio
OTCS: Ocular trauma classification system
OTS: Ocular trauma score
PVR: Proliferative vitreous retinopathy
RAPD: Relative afferent pupillary defect
RD: Retinal detachment
TON: Traumatic optic neuropathy
VA: Visual acuity
VH: Vitreous hemorrhage
