# Supplementary material for: Explosive eye injuries: characteristics, traumatic mechanisms, and prognostic factors for poor visual outcomes
Source: Mil Med Res. 2023 Jan 12;10:3. doi: 10.1186/s40779-022-00438-4 (PMC9835351; doi:10.1186/s40779-022-00438-4)
Supplement: Supplementary file 1 — Additional file 1: Table S1. General features of the explosive eye injuries (total of 1115 patients). Fig. S1. Distribution of the number of patients and the proportions of male patients and bilateral eye injuries in different age groups. [file 40779_2022_438_MOESM1_ESM.pdf]

Table S1 General features of the explosive eye injuries (total of 1115 patients)

Characteristics	No. of patients [*n*(%)]	Specification
**Gender**		
Male	986 (88.43)	262 adolescents (26.57%), (1 − 76) years, median 30 years
Female	129 (11.57)	40 adolescents (31.01%), (3 − 75) years, median 31 years
**Age groups (years)**		
Adolescents (≤ 18)	302 (27.09)	262 males (86.75%), 47 biocular injuries (15.56%)
1 − 6	53 (4.76)	40 males (75.47%), 5 biocular injuries (9.43%)
7 − 12	132 (11.84)	114 males (86.36%), 21 biocular injuries (15.91%)
13 − 18	117 (10.49)	108 males (92.31%), 21 biocular injuries (17.95%)
Adults	813 (72.91)	724 males (89.05%), 287 biocular injuries (35.30%)
19 − 39	567 (50.85)	510 males (89.95%), 201 biocular injuries (35.45%)
40 − 59	219 (19.64)	194 males (88.58%), 82 biocular injuries (37.44%)
≥ 60	27 (2.42)	20 males (74.07%), 4 biocular injuries (14.81%)
**Laterality of injured eyes**		
Bilateral	334 (29.96)	There were 558 right eyes and 557 left eyes in the 1449 injured eyes
**Identity**		
Workers	386 (34.62)	179 miners (46.37%), 13 construction workers, 5 mechanics, others unknown
Students	246 (22.06)	
Farmers	153 (13.72)	
Office staff	60 (5.38)	
Preschool children	49 (4.39)	
Commercial and service staff	46 (4.13)	
Drivers	16 (1.44)	
Others or unknown	159 (14.26)	
**Seasons**		
Spring (March − May)	242 (21.70)	
Summer (June − August)	210 (18.83)	

Characteristics	No. of patients [*n*(%)]	Specification
Fall (September – November)	186 (16.68)	
Winter (December – February)	477 (42.78)	
**Explosives**		
Fireworks or firecrackers	392 (35.16)	Some exploding firecrackers put in containers such as glass bottles or pen caps
Mine gases	191 (17.13)	
Detonators	133 (11.93)	
Containers	110 (9.87)	Beer bottle (40 cases), can, thermos flask or cup, bucket, oxygen tank, containers with peracetic acid, mannitol, etc.
Light bulb or tube	33 (2.96)	
Battery	31 (2.78)	Including accumulator storage battery of electromobile, some due to man-made hammering or burning batteries
Tire	25 (2.24)	
Firearms (shell, bomb, bullet)	22 (1.97)	Military activities, hunting, some cases hammering of sawing bullets
Other electric machinery	14 (1.26)	Immersion heater, electric welding, perforating gun, nail gun, dial instrument, mosquito-repellent incense, etc.
Pipe or pump	11 (0.98)	Hot water pipe, hydraulic pump, etc.
Boiler	11 (0.98)	
Lighter	9 (0.81)	
Pressure cooker	6 (0.54)	
Others or unknown	127 (11.39)	Fuel, such as gas, petrol, calcium carbide, saltpeter, TNT, sulfur, lime, etc.
**Multiple other injuries**		
Coma following explosion	22 (1.97)	
Craniocerebral injury	83 (7.44)	
Limb injuries	155 (13.90)	

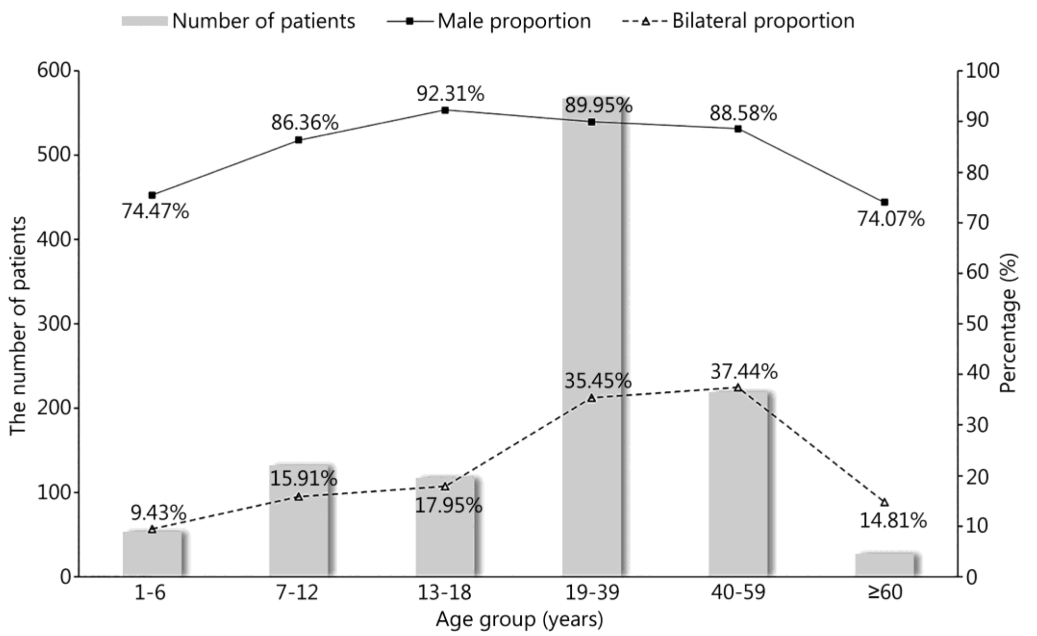

Fig. S1 Distribution of the number of patients and the proportions of male patients and bilateral eye injuries in different age groups. The youth adults (19 – 39 years) had the most eye injuries, the teenager (13 – 18 years) had the highest proportion of male, and the middle-aged adults (40 – 59 years) had the highest proportion of binocular injury
